# Draft Genome Sequences of Three *Listeria monocytogenes* Isolates from Foods in China

**DOI:** 10.1128/genomeA.00220-17

**Published:** 2017-04-20

**Authors:** Xudong Su, Guojie Cao, Dai Kuang, Jianmin Zhang, Yi Chen, Marc Allard, Eric Brown, Xianming Shi, Jianghong Meng, Xuebin Xu

**Affiliations:** a Department of Food Science & Technology, Shanghai Jiao Tong University, Shanghai, China; b Center for Food Safety and Applied Nutrition, Food and Drug Administration, College Park, Maryland, USA; c College of Veterinary Medicine, South China Agricultural University, Guangzhou, China; d Department of Nutrition and Food Science, University of Maryland, College Park, Maryland, USA; e Shanghai Municipal Center for Disease Control and Prevention, Shanghai, China

## Abstract

*Listeria monocytogenes* is a foodborne pathogen of global concern because of the high mortality rate among patients. The draft genome sequences of three *L. monocytogenes* strains isolated from foods are reported here. The availability of these genomes should provide useful information on the genomic diversity of *L. monocytogenes* isolated from foods in China.

## GENOME ANNOUNCEMENT

*Listeria monocytogenes* is an important foodborne pathogen causing severe illness, such as septicemia and meningitis, among pregnant women, elderly people, and immunocompromised individuals (1, 2). *L. monocytogenes* is ubiquitous and has been reportedly isolated from a wide range of foods, including meat, poultry, dairy products, fruit, and vegetables (3, 4). Whole-genome sequencing has become an essential tool for investigating the evolution, diversity, and pathogenicity of foodborne bacterial pathogens (5–9). The draft genome sequences of three *L. monocytogenes* strains from foods in the Shanghai area of China are reported here.

Genomic DNA was isolated from overnight cultures and sequenced using MiSeq (Illumina, San Diego, CA) to obtain draft genomes. Genomic data were assembled using SOAP*denovo* version 2 (10), including the serotype 1/2c strain SHL12-2 (16 contigs, 3.01 Mb, 583,457 bp *N*_50_ contig size, and 2,978 identified genes), serotype 1/2a strain SHL12-22 (20 contigs, 3.02 Mb, 506,776 bp *N*_50_ contig size, and 2,943 identified genes), and serotype 1/2a strain SHL13-12 (17 contigs, 2.99 Mb, 524,215 bp *N*_50_ contig size, and 2,884 identified genes). These sequences were annotated using the NCBI Prokaryotic Genome Annotation Pipeline (11).

### Accession number(s).

These three draft genome sequences have been deposited in GenBank under the accession numbers LRTW00000000, LRTX00000000, and LRTY00000000.
